# dLagM: An R package for distributed lag models and ARDL bounds testing

**DOI:** 10.1371/journal.pone.0228812

**Published:** 2020-02-21

**Authors:** Haydar Demirhan

**Affiliations:** Mathematical Sciences Discipline, School of Science, RMIT University, Melbourne, Victoria, Australia; Univerza v Mariboru, SLOVENIA

## Abstract

In this article, we introduce the R package dLagM for the implementation of distributed lag models and autoregressive distributed lag (ARDL) bounds testing to explore the short and long-run relationships between dependent and independent time series. Distributed lag models constitute a large class of time series regression models including the ARDL models used for cointegration analysis. The dLagM package provides a user-friendly and flexible environment for the implementation of the finite linear, polynomial, Koyck, and ARDL models and ARDL bounds cointegration test. Particularly, in this article, a new search algorithm to specify the orders of ARDL bounds testing is proposed and implemented by the dLagM package. Main features and input/output structures of the dLagM package and use of the proposed algorithm are illustrated over the datasets included in the package. Features of dLagM package are benchmarked with some mainstream software used to implement distributed lag models and ARDLs.

## Introduction

Distributed lag models (DLMs) constitute a class of regression models which include lags of explanatory time series as independent variables. They provide a flexible way of involving independent series in dynamic regression models. DLMs are dynamic models in the sense that they include past values of both dependent and independent series. Although DLMs are mostly used in econometric analyses, because they provide regression-like analysis for time series data, they are also frequently utilized in a wide range of fields including energy, marketing, agriculture, epidemiology, and environmental studies [1]. In recent literature, Zhang et al. [2] apply DLMs to analyze gestational diabetes mellitus and maternal weekly air pollution exposure. Heaton et al. [3] model individualized effects of heat on health using DLMs. Nerudova and Dobransch [4] use time-varying variable in the DLM setting to analyze the value-added tax gap in the European Union. Nothdurft and Engel [5] utilize the DLMs to investigate the effect of species mixing on productivity and climate-related resistance. Guler et al. [6] propose biased estimators for the parameters of DLMs. Mulchandani et al. [7] employ advertising effectiveness in the banking sector of India using a particular DLM, namely the Koyck model. Ozsayin [8] implements a Koyck model approach to analyze the relationship between the production and the price of cow milk. Berk [9] uses the Koyck model to investigate the association between sunflower production and its price in Turkey. Alotaish et al. [10] use another type of DLMs, namely autoregressive distributed lag (ARDL) models, to investigate the role of government expenditure and financial development in environmental degradation. Csereklyei et al. [11] analyze the relationship between the amount of wind and solar energy generation and wholesale electricity prices in Australia.

One of the important application areas of DLMs is cointegration analysis where short and long-run relationships between series are investigated. ARDL bounds testing of Pesaran et al. [12] is a popular cointegration analysis method based on DLMs in the literature. In recent literature, Zhang et al. [13] use ARDL models to analyze the relationship between economic growth, carbon emissions, and energy consumption in the main grain-producing areas of China between 1996 and 2015. Ullah et al. [14] figure out the causal relationship between the agricultural ecosystem and carbon dioxide (CO2) emissions in Pakistan using the data between 1972 and 2014. Zafeiriou et al. [15] focus on agricultural carbon emissions and income per capita in the agriculture sector of the European Union by using nonlinear ARDL models. Pal and Mitra [16] implement autoregressive DLMs to analyze the relationship between diesel and soybean prices in the USA. Bello et al. [17] employ DLMs for modelling the longitudinal effects of chemicals to figure out the time-delayed effect of an exposure on an outcome. Huang et al. [18] investigate the association between cardiovascular mortality and temperature using nonlinear DLMs. Zhu et al. [19] analyze the relationship between air pollution and years of life lost using nonlinear DLMs. Baek et al. [20] analyze the associations between built environments and health based on spatial information using a hierarchical version of DLMs. DLMs have frequently been used by a very general group of practitioners in recent literature. The list of studies utilizing the DLMs in practice can easily be extended.

Considering their usefulness, flexibility, and broad audience in a wide range of fields, implementation of DLMs and ARDL bounds testing by open-source research software in a user-friendly way is of critical importance. The most comprehensive R package developed for the implementation of both linear and nonlinear DLMs is dlnm [21, 22]. The dlnm package takes into account nonlinear and delayed relationships between dependent and independent series, simultaneously. Huang et al. [18] and Zhu et al. [19] employ the dlnm package for fitting nonlinear DLMs in their studies. Although this approach provides a powerful solution for fitting nonlinear DLMs, it requires a good understanding of the idea of cross-basis functions and their construction with dlnm for linear DLMs. This is not an easy task for general practitioners. Another package developed to implement DLMs is nardl [23]. The nardl package is specifically focused on the implementation of the nonlinear cointegrating autoregressive distributed lag model proposed by Shin and Greenwood-Nimmo [24]. The package dynlm [25] has just one main function to fit dynamic linear models by preserving time series characteristics. A recently published R package, dynamac [26] is specifically designed to simulate the effect of some independent series on dependent by dynamic simulations and run the ARDL bounds test of Pesaran et al. [12]. dynamac has some functions for fitting the ARDL models and plotting and simulating results. However, it does not include other specific DLMs such as Koyck model and provides any function for the specification of lag orders. It allows user to define lags, differences, and lag-differences of the series. The software EViews [27] is the main player in econometric analyses. However, this very powerful software is not freely available and requires familiarity with its user-interface to fit models and draw inferences.

In this article, we describe our R package dLagM version 1.0.21 that implements DLMs and ARDL bounds testing approach of Pesaran et al. [12]. Due to its simplicity in the user interface, the package does not require high-level programming skills or a strong understanding of various data structures. It provides a straightforward interface for model fitting in the open-source environment R. In general, since dLagM gets the dataset, lag orders, and a general model formula and creates the required lags and differences for specific models, users do not need to specify the differences for the implemented models. In this sense, it has a high utility and is quite useful for many researchers from different fields without powerful statistical knowledge and background. One of our main contributions is that we propose and implement a new search algorithm for the specification of lag orders in ARDL bounds testing. Specifying an optimal lag order for the ARDL bounds test has crucial importance on the test results since it impacts all the inferences on the long-run and short-run relationships between the dependent and independent series. This new algorithm brings in a significant improvement on the computation time to find an appropriate lag order for the test. Therefore, it is possible to search over larger sets of possible models for the optimal lag orders. As another main contribution, the package provides a function to investigate the significance of the signals in time series data by using the method proposed by Gershunov, et al. [28]. To the best of our knowledge, there is no other readily available, open-source software implementation of this approach. Lastly, the package implements recently proposed accuracy measures based on relative absolute errors by Chen at al. [29] in addition to the mainstream measures. Overall, we introduce a user-friendly package that makes use of distributed lag models and ARDL bounds testing approach readily available for practitioners from non-statistics backgrounds. Input and output structures of the functions in dLagM are fully compatible with the general modelling framework of R. In this sense, it targets a broad audience of all levels of competence with R.

In Section 2, we first illustrate testing the significance of signals before going on with fitting DLMs, contextual characteristics of dLagM in fitting DLMs, producing forecasts for dependent series, and computing the goodness-of-fit measures. In Section 3, we give a brief methodology for the ARDL bounds testing and explain its implementation with the dLagM package. We describe our algorithm to specify lag orders for the test and illustrate the implementation of the test with a dataset available in the package. The last section is devoted to discussion.

## Main functionality of dLagM package

The dLagM package is published on the Comprehensive R Archive Network (CRAN). The following codes are to install and load the package to the R environment:

install.packages(“dLagM”)

library(“dLagM”)

The dLagM package provides a Monte Carlo approach to test if the signals in two series are significantly different from the white noise, fits finite, polynomial, Koyck’s, and autoregressive DLMs with rolCorPlot(), dlm(), polyDlm(), koyckDlm(), and ardlDlm() functions, respectively; and it implements ARDL bounds test by ardlBound() function. The dlm() and ardlDlm() functions can handle multiple independent series and they provide the flexibility of removing specific lags of independent and/or dependent series from finite and autoregressive DLMs.

### Testing the significance of signals

When we work with relatively short and low-frequency time series including linear trends and seasonality, there is the risk of taking the white noise as signal [28]. Therefore, it is useful to test the significance of signals before moving onto further analyses such as DLMs and the ARDL bounds testing. To test the existence of signal against noise, Gershunov et al. [28] proposed a Monte Carlo test approach based on the standard deviations of rolling correlations. In this test, standard deviations of empirical rolling correlations are compared to those of two correlated white noise series replicated many times with the same magnitude of correlation as the empirical series [28]. Higher or lower variability in the standard deviations of rolling correlations than that of two white noise series suggests that the series is not dominated by other characteristics such as trend or seasonality; hence, the correlation between the series is physical. This procedure does not test the existence of a precedence relationship between the series. It rather tests whether the correlation patterns within predefined time windows are significantly different from those in the white noise series. dLagM includes two functions to implement this approach. To the best of our knowledge, there is no readily available software to implement this test other than dLagM.

Rolling correlations are generated and plotted by the following function:

rolCorPlot(x, y, width, start = 1, level = 0.95, main = NULL, SDtest = TRUE)

which implements the Monte Carlo test approach utilizing the roll_cor() function from the roll package [30] and plots the rolling correlations between x and y for the given window lengths by width. It also plots the average rolling correlation over the window lengths after smoothing with nonlinear running median filter by runmed() function of stats package [31] along with level% confidence limits. To test the significance of signal, SDtest is set to TRUE. The following function

sdPercentiles(n = 150, cor = 0.5, width = 5, N = 500, percentiles = c(.05, .95))

produces one-tailed test limits for this approach for the given length n, correlation cor, width and percentiles. The number of Monte Carlo replications is specified by N. If the standard deviations of running correlations between empirical series are outside these limits, we conclude that the signal in the series is significantly more or less variable than expected from noise [28]. sdPercentiles() can be used independently of the rolCorPlot() to produce the limits for any pair of series.

For illustrative purposes, we use the dataset seaLevelTempSOI composed of monthly mean global sea level (GMSL, compared to 1993-2008 average) [32], monthly mean global land-ocean temperature anomalies (1951-1980 as a baseline period) [33], and monthly Southern Oscillation Index (SOI) [34] series between July 1885 and June 2013. We use the last 8 years of the GMSL and temperature anomalies series to demonstrate the usage of the function with the following code chunk.

data(seaLevelTempSOI)

level.ts <- ts(seaLevelTempSOI[1499:1595,1], start = c(2005,5), freq = 12)

temp.ts <- ts(seaLevelTempSOI[1499:1595,2], start = c(2005,5), freq = 12)

rolCorPlot(y = level.ts, x = temp.ts, width = c(3, 5, 7, 11), level = 0.95,

main = “Rolling correlations between sea levels and temperature”,

SDtest = TRUE)

The output includes the rolling correlations for each width in $rolCor, the average rolling correlations filtered by running median filter against outliers in $rolcCor.avr.filtered, the unfiltered average rolling correlations in $rolcCor.avr.raw, standard deviations of rolling correlations for each width in rolCor.sd, Pearson correlation between two series in $rawCor, the percentiles of MC distribution of standard deviations of rolling correlations as the test limits in $sdPercentiles, and the standard deviations of rolling correlations for each width along with level% and (1 − level)% limits in $test. The following section of the output shows the test results:

$test

Width SDrolCor     95%     5%

1   3 0.7431643 0.7486708 0.6505622

2   5 0.5806556 0.5548243 0.4171213

3   7 0.4800409 0.4715809 0.3172123

4  11 0.3975792 0.3733404 0.2047842

**Fig 1:**
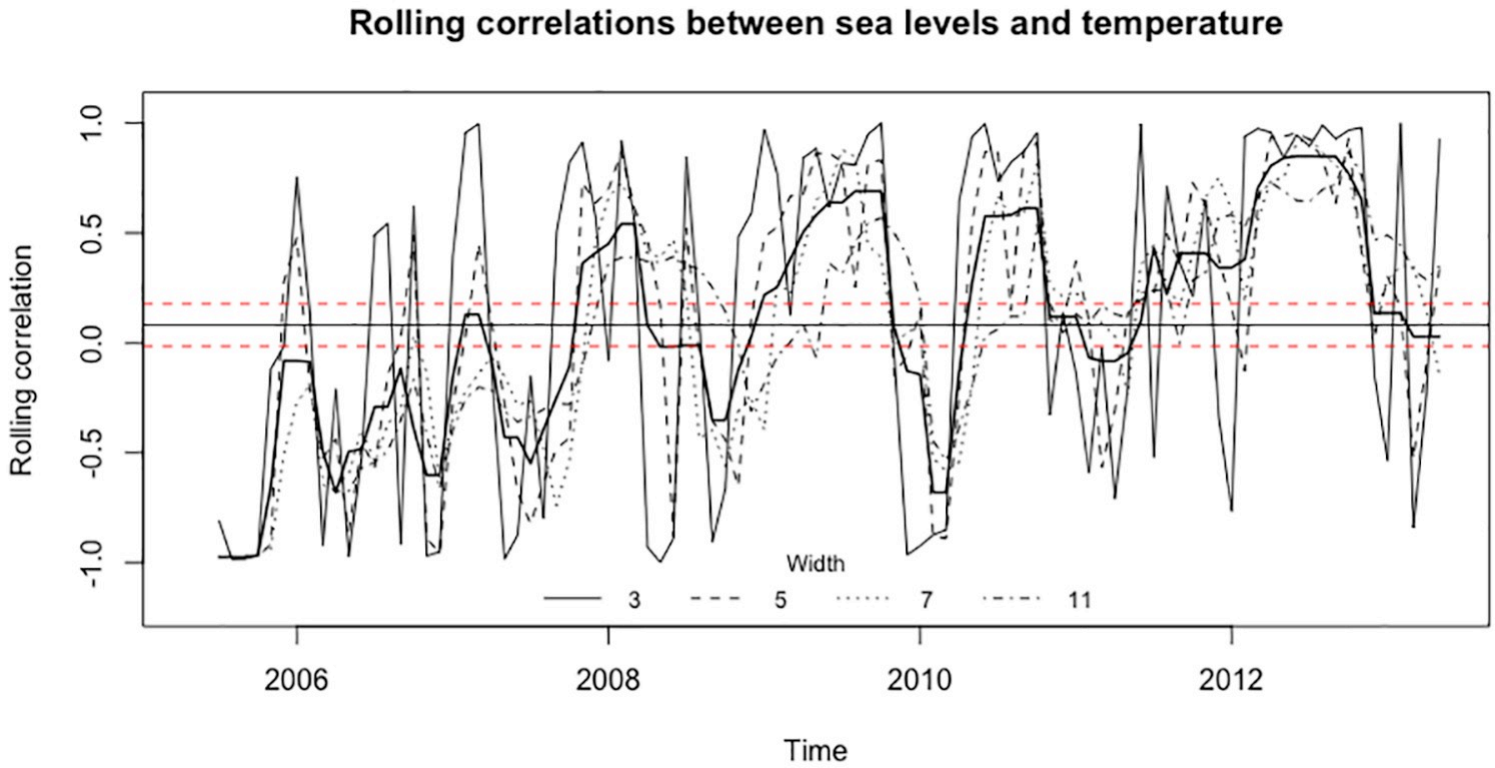
Rolling correlations between the monthly GMSL and mean land-ocean temperature series.

The rolling correlation standard deviations (SDs) are outside the limits for the widths 5, 7, and 11. However, the SD for the width of 3 is inside the limits. Thus, the signal between the GMSL and the temperature series is significant for wider window lengths; hence, the rolling correlations between series are not superfluous. Fig 1 shows the plot generated by the rolCorPlot function.

In Fig 1, the dashed red lines show the limits of the 95% confidence interval for the mean of the average rolling correlations over the time points, which is shown by the horizontal solid line. The bold, solid line shows the average rolling correlation over the widths. A line is plotted to show the rolling correlations for each width. In accordance with the test results, rolling correlations for widths of 5, 7, and 11 vary more than those for the width of 3. High rolling correlations are recorded between 5 and 7-years-apart sea level and the temperature observations between 2009 and 2010, after both 2010 and 2012 which are significantly different from the rolling correlations between two independent white noise series.

### Finite distributed lag models

DLMs are used to model the current and delayed effects of an independent {*X*_*t*_} series on a dependent {*Y*_*t*_} series, where *i* = 1, 2, …, *n*. The infinite linear DLM is written as follows:

$$\begin{array}{c} Y_{t}=\alpha +\sum_{s=0}^{\infty }\beta_{s}X_{t-s}+\epsilon_{t}, \end{array}$$

where *α* shows the overall mean effect and *β*-parameters represent the lag weights. *β*_0_ shows the effect of independent series and *β*_*s*_ shows the effect of lags of the independent series for *s* = 1, 2, …. The parameter *ϵ*_*t*_ is the stationary error term for the time point *t* with zero mean and constant variance [35]. When the summation is bounded above by *q*, the DLM turns out to be a finite linear DLM of order *q*. Notice that the number of available {*Y*_*t*_, *X*_*t*_} pairs for estimation of model parameters is *n* − *q*. A weakness of finite DLMs is the existence of multicollinearity with increasing lag orders due to including multiple lags of the same series in the model.

To produce parameter estimates and their significance tests, we employ the dlm() function, which is capable of fitting multiple independent variables with predefined subsets of their lags. Call to the function dlm() is as follows:

dlm(formula,  data,  x,  y,  q,  remove)

The dependent and independent series can be fed into the dlm() function either using formula and data arguments or x and y arguments. If there is only one independent series, use of x and y arguments provides the straightforward way. When there are multiple independent series, all the dependent and independent series should be included in the argument data of the class data.frame and the model should be passed on by the argument formula, which is a usual formula object of R. There is no need to include the lags of independent series in the formula since they are automatically generated by dlm(). The column names of data corresponding to the independent and dependent series must be the same as those in the formula.

The argument q shows the finite lag length. To drop predefined lags of some independent series from the model, we use the remove argument. Each element of the list remove shows particular lags that will be removed from each independent series. To demonstrate the constriction of the remove argument, suppose we arbitrarily have the dependent series **Y** and independent series **X** and **Z** with formula = Y ∼ X + Z and q = 5. Then, if remove is set to NULL, the fitted model will include all lags from 1 to 5 for both **X** and **Z** series. To arbitrarily remove the lags 2, 3, and 4 of **X** and 2 and 4 of **Z** series, remove should be defined as follows:

> remove <- list(X = c(2,3,4), Z = c(2,4))

Notice that it is possible to fit a model with different lag lengths for each independent series by removing the desired lags using the remove argument and the main series can be removed from the model when 0 is included within the elements of remove.

To illustrate the implementation of the DLMs with dLagM package, we use the GMSL and temperature anomalies series from seaLevelTempSOI dataset. First, we load the dataset to R environment, fit a finite DLM with lag length 7, which gives the minimum BIC, and display the model output with the following code chunk. In accordance with R’s user interface, the model summary, AIC, BIC, residuals, fitted values, and coefficients are displayed by summary(), AIC(), BIC(), residuals(), fitted(), and coef() functions, respectively.

> dlmFit1 <- dlm(formula = GMSL ~ LandOcean,

          data = seaLevelTempOSI, q = 7)

> summary(dlmFit1)

Residuals:

   Min    1Q Median   3Q   Max

-19.8515 -2.5781 0.1053 2.5427 19.9983

Coefficients:

        Estimate Std. Error t value Pr(>|t|)

(Intercept)  -0.008943 0.101086 -0.088 0.9295

LandOcean.t   0.746159 1.153242 0.647  0.5177

LandOcean.1  -0.176851 1.158004 -0.153 0.8786

LandOcean.2  -1.618818 1.154572 -1.402 0.1611

LandOcean.3  -1.977914 1.151842 -1.717 0.0861 .

LandOcean.4  -2.488326 1.151439 -2.161 0.0308 *

LandOcean.5  -2.288856 1.154280 -1.983 0.0475 *

LandOcean.6  -2.248660 1.157732 -1.942 0.0523 .

LandOcean.7  -2.441134 1.153526 -2.116 0.0345 *

---

Signif. codes: 0 ‘***’ 0.001 ‘**’ 0.01 ‘*’ 0.05 ‘.’ 0.1 ‘ ’ 1

Residual standard error: 4.028 on 1579 degrees of freedom

Multiple R-squared: 0.01724, Adjusted R-squared: 0.01226

F-statistic: 3.463 on 8 and 1579 DF, p-value: 0.0005765

AIC and BIC values for the model:

    AIC    BIC

1 8942.719 8996.422

Although the model is significant at 5% level of significance (p-value: 0.0005765), it gives a poor fit in terms of the adjusted R-squared of 1.2%. The coefficients for the lags greater than 2 are significant either at 5% or 10% level. The distribution of residuals is nearly symmetric. Then, we remove independent series (with 0 in the remove), its first and second lags, and the intercept (with -1 in the model formula) from the model only to illustrate the use of the remove argument.

> remove = list(LandOcean = c(0,1,2))

> dlmFit2 <- dlm(formula = GMSL ~ -1 + LandOcean,

         data = seaLevelTempSOI, q = 6, remove = remove)

> summary(dlmFit2)

Residuals:

   Min    1Q  Median    3Q   Max

-20.0704 -2.6089  0.0976 2.5265 20.1688

Coefficients:

       Estimate Std. Error t value Pr(>|t|)

LandOcean.3  -2.072  1.109 -1.868  0.0619 .

LandOcean.4  -2.266  1.116 -2.030  0.0425 *

LandOcean.5  -2.147  1.116 -1.923  0.0547 .

LandOcean.6  -2.480  1.108 -2.237  0.0254 *

---

Signif. codes: 0 ‘***’ 0.001 ‘**’ 0.01 ‘*’ 0.05 ‘.’ 0.1 ‘ ’ 1

Residual standard error: 4.034 on 1585 degrees of freedom

Multiple R-squared: 0.01345, Adjusted R-squared: 0.01096

F-statistic: 5.4 on 4 and 1585 DF, p-value: 0.0002555

AIC and BIC values for the model:

    AIC    BIC

1 8947.933 8974.788

With this model, we get similar results on the significance of the coefficients and the reduced model. Note that since R uses a different formula for R-squared when there is no intercept in the model, R-squared values are not comparable. Also, these outputs are given neither to compare two models nor to draw decisive inferences on the relationship between the temperature anomalies and the sea level series. The intention here is to illustrate the usage of the functions. The residuals, fitted values, and coefficients of the model dlmFit2 are obtained by residuals(dlmFit2), fitted(dlmFit2), and coef(dlmFit2), respectively.

It is possible to impose a polynomial shape on the lag weights to fit a polynomial DLM that is fitted by the following call to the function polyDlm():

polyDlm(x, y, q, k, show.beta = TRUE)

Currently, there is only one independent variable allowed in polyDlm() function. The arguments x and y are used to send independent and dependent series into the function, respectively. The arguments q and k are used to specify the lag and polynomial orders, respectively. To show the values of actual *β* parameters and the results of their significance tests, show.beta needs to be set to TRUE.

### Koyck distributed lag models

Another approach to specify lag weight is to impose a geometrically decreasing pattern on the lag weights. The following equation shows the general form of a geometric DLM with geometrically decreasing lag weights *β*_*s*_ = *βϕ*^*s*^:

$$\begin{array}{c} Y_{t}=\alpha +\beta \left(X_{t}+\phi X_{t-1}+\phi^{2}X_{t-2}+\phi^{3}X_{t-3}+\cdots \right)+\epsilon_{t}. \end{array}$$

The implementation of geometric DLMs is not practical due to the infinite number of parameters and nonlinearity of the model. Koyck transformation is an efficient way of dealing with these drawbacks of the geometric DLMs. The Koyck form of the geometric DLM is as follows [35]:

$$\begin{array}{c} \begin{array}{cc} Y_{t} & =\alpha \left(1-\phi \right)+\phi Y_{t-1}+\beta X_{t}+\left(\epsilon_{t}-\phi \epsilon_{t-1}\right)\\ & =\delta_{1}+\delta_{2}Y_{t-1}+\delta_{3}X_{t}+\nu_{t}. \end{array} \end{array}$$

As the result, we get a finite DLM after the Koyck transformation. Notice that the first lag of the dependent series *Y*_*t*−1_ and the error term *ν*_*t*_ are correlated with each other and the error term *ν*_*t*_ depends on both *ϵ*_*t*_ and *ϵ*_*t*−1_. Therefore, we use instrumental variables approach to estimate model parameters in dLagM [36]. The function koyckDlm() implements the instrumental variables estimation. The call to the function is as follows:

koyckDlm(x, y)

Currently, only one independent variable is allowed in koyckDlm() function. The arguments x and y are used to send the independent and dependent series into the function, respectively. Diagnostics for the Koyck DLM are displayed by

summary(KoyckFit, diagnostics=TRUE)

given the fitted model is KoyckFit. All the diagnostic tests are implemented by the package AER [36]. In the Weak Instruments line of the output, an F test of the first stage regression for weak instruments is displayed. In the second line, the Wu-Hausman test is displayed for the significance of the correlation between an explanatory variable and the error term (endogeneity). In the Koyck DLM, the significance of the correlation between the error term and the lagged dependent variable is not clear. The Wu-Hausman test, which compares the consistency of instrumental variable estimates to ordinary least squares estimates, provides a way for testing the significance of the correlation between the error term and the dependent series. If the endogeneity is present, the instrumental variable estimator is concluded to be consistent while the ordinary least squares estimator is inconsistent. But this test should be used cautiously with small samples due to its asymptotic validity [37].

We fit the Koyck model with GMSL and land-ocean temperature anomalies series and display the summary statistics with the following code chunk:

> KoyckFit <- koyckDlm(x = seaLevelTempSOI$LandOcean, y = seaLevelTempSOI$GMSL)

> summary(KoyckFit, diagnostics=TRUE)

Residuals:

    Min     1Q   Median     3Q     Max

-16.74923 -1.75048 -0.02326 1.68498  11.97262

Coefficients:

       Estimate Std. Error t value Pr(>|t|)

(Intercept) 0.008036 0.070254 0.114 0.909

Y.1      0.733753 0.017582 41.734 <2e-16 ***

X.t     -2.553163 3.607947 -0.708 0.479

---

Signif. codes: 0 ‘***’ 0.001 ‘**’ 0.01 ‘*’ 0.05 ‘.’ 0.1 ‘ ’ 1

Residual standard error: 2.805 on 1591 degrees of freedom

Multiple R-Squared: 0.5297, Adjusted R-squared: 0.5291

Wald test: 906.6 on 2 and 1591 DF, p-value: < 2.2e-16

Diagnostic tests:

         df1  df2    statistic    p-value

Weak instruments 1 1591  70.3332073 1.088953e-16

Wu-Hausman     1 1590   0.9925055 3.192824e-01

                 alpha    beta    phi

Geometric coefficients: 0.03018417 -2.553163 0.7337525

We have a significant model and a coefficient with lower adjusted R-squared. There is no significant endogeneity by the Wu-Hausman test and no significant weak instruments problem by the Weak Instruments test.

### Autoregressive distributed lag models

The autoregressive DLMs are employed to implement a regression analysis between a dependent series and *k* number of independent series. When there is only one independent series, an ARDL model of orders *p* and *q* is denoted by ARDL(*p*, *q*), which consists of *p* lags of independent and *q* lags of dependent series. The lags of the dependent series make the model autoregressive. When the number of lags of *i*th independent series is shown by *p*_*i*_, *i* = 1, …, *k*, we write the ARDL model as in Eq (1):

$$\begin{array}{c} \begin{array}{cc} Y_{t}=\mu_{0} & +\sum_{i=1}^{k}\beta_{0i}X_{ti}+\beta_{1i}X_{(t-1)i}+\cdots +\beta_{pi}X_{(t-p_{i})i}\\ & +\gamma_{1}Y_{t-1}+\cdots +\gamma_{q}Y_{t-q}+e_{t}. \end{array} \end{array}$$ (1)

where *μ*_0_ is the constant, *Y*_*t*_ and *X*_*ti*_ are respectively dependent and *i*th independent series, *p*_*i*_ is the lag order of *i*th independent series, *q* is the autoregressive order of the model, and *e*_*t*_ shows the innovations.

To implement autoregressive DLMs, the call to the ardlDlm() function is

ardlDlm(formula = NULL, data = NULL, x = NULL, y = NULL, p = 1, q = 1, remove = NULL)

The same convention as the dlm() function is followed to feed the data and the model into the ardlDlm() function. The argument p shows the values of lag orders such that *p_1_* = *p_2_* = ⋯ = *p_k_* = *p*. The argument q shows the value of autoregressive orders of the model. This makes the call to the function easier when all lag orders are equal. When the lag order of each independent series differs, we employ remove argument to remove unwanted lags. It is also used to remove some lags of dependent series, simultaneously. In this sense, it is very flexible in fitting parsimonious models. It has the following list structure:

remove <- list(p = list(), q = c())

Each element of the list p shows particular lags that will be removed from each independent series. To remove the main series from the model or to fit an ARDL(0,q) model, we include 0 within the elements of p. The element q is a vector showing the autoregressive lags of dependent series to be removed. Following the example given for DLM implementation, to remove the main series of **X** and **Z**, the second lag of **X** and the first autoregressive lag of **Y** from the model, we define remove as follows:

remove <- list(p = list(X = c(0,2), Z = c(0)), q = c(1)).

Note that the generic functions mentioned for dlm() function works for all the models fitted by dLagM.

To demonstrate the use of the function with multiple independent series, we use all three series available by the seaLevelTempSOI dataset available in the package. The following code chunk fits an ARDL model for the GMSL (*Y*) series with land-ocean temperature anomalies (*X*_1_) and SOI (*X*_2_) series. For demonstration purposes, we arbitrarily take *p*_1_ = 2, *p*_2_ = 1, *q* = 4 and fit the ARDL model.

> formula1 <- GMSL ~ LandOcean + SOI

> remove <- list(p = list(SOI = c(2)))

> ARDLfit1 <- ardlDlm(formula = formula1, data = seaLevelTempSOI, p = 2, q = 4, remove = remove)

> summary(ARDLfit1)

Residuals:

    Min    1Q Median   3Q   Max

-11.0820 -1.2365 -0.0346 1.1360 7.5509

Coefficients:

        Estimate Std. Error t value Pr(>|t|)

(Intercept)   0.003920 0.049067 0.080 0.93633

LandOcean.t   1.129695 0.533647 2.117 0.03442 *

LandOcean.1   0.291991 0.545341 0.535 0.59243

LandOcean.2  -1.369952 0.531626 -2.577 0.01006 *

SOI.t      -0.010608 0.006061 -1.750 0.08027 .

SOI.1      0.002837 0.006078  0.467 0.64071

GMSL.1      0.951282 0.024389 39.004 < 2e-16 ***

GMSL.2      0.081073 0.028575  2.837 0.00461 **

GMSL.3     -0.745129 0.028568 -26.083 < 2e-16 ***

GMSL.4      0.240132 0.024381  9.849 < 2e-16 ***

---

Signif. codes: 0 ‘***’ 0.001 ‘**’ 0.01 ‘*’ 0.05 ‘.’ 0.1 ‘ ’ 1

Residual standard error: 1.957 on 1581 degrees of freedom

Multiple R-squared: 0.7718,Adjusted R-squared: 0.7705

F-statistic: 594 on 9 and 1581 DF, p-value: < 2.2e-16

The coefficient related to *X*_1_ series and its second lag, and all four lags of the *Y* series are significant at the 5% significance level. Also, *X*_2_ series is significant at the 10% level. The model is significant the 5% level with a p-value less than 2.2e-16. We can take a further step and remove insignificant coefficients from the model as follows:

> remove <- list(p = list(LandOcean = c(1), SOI = c(1,2)))

> ARDLfit2 <- ardlDlm(formula = formula1, data = seaLevelTempSOI, p = 2, q = 4, remove = remove)

> summary(ARDLfit2)

Residuals:

    Min   1Q Median  3Q  Max

 -11.060 -1.237 -0.030 1.159 7.504

Coefficients:

       Estimate Std. Error t value Pr(>|t|)

(Intercept)   0.003949 0.049043 0.081 0.93583

LandOcean.t   1.185116 0.521855 2.271 0.02328 *

LandOcean.2  -1.320628 0.519258 -2.543 0.01108 *

SOI.t      -0.008862 0.004722 -1.877 0.06074 .

GMSL.1      0.951720 0.024314 39.142 < 2e-16 ***

GMSL.2      0.080261 0.028509  2.815 0.00493 **

GMSL.3     -0.744486 0.028536 -26.089 < 2e-16 ***

GMSL.4      0.239964 0.024349  9.855 < 2e-16 ***

---

Signif. codes: 0 ‘***’ 0.001 ‘**’ 0.01 ‘*’ 0.05 ‘.’ 0.1 ‘ ’ 1

Residual standard error: 1.956 on 1583 degrees of freedom

Multiple R-squared: 0.7717, Adjusted R-squared: 0.7707

F-statistic: 764.4 on 7 and 1583 DF, p-value: < 2.2e-16

According to this model, the previous year’s Southern Oscillation Index, temperature anomaly recorded in the current year and two years before, and sea levels of one, two, three, and four years before have a significant impact on the sea level in a given year. The model is significant at the 5% level (*P* < 2.2*e* − 16), the adjusted R-squared value is at a moderate to high level with 77.07%. A model selection procedure needs to be applied to find the optimum model for this data. Here, we aim to explain the use of the arguments of the ardlDlm() function.

### Forecasting with distributed lag models

For all the models implemented by the dLagM package, forecasts are generated by the generic forecast() function:

forecast(model, x, h = 1, interval = FALSE, level = 0.95, nSim = 500)

The argument model is a fitted model by one of the functions of dLagM package. x includes the new data for independent series with a forecast horizon of h. The (1 − level)% prediction intervals for forecasts are obtained by the Monte Carlo approach using a Gaussian error distribution with zero mean and empirical variance of the dependent series if interval is set to TRUE.

To illustrate the use of the function, the following code chunk generates 3 months ahead sea level forecasts with 95% prediction intervals using the fitted ARDL model ARDLfit2.

> x <- matrix(c(0.06, 0.07, 0.08, 13.9, 8.1, -0.5), nrow = 2, ncol = 3, byrow = T)

> forecast(model = ARDLfit2, x = x, h = 3, interval = TRUE)$forecasts

   95% LB  Forecast  95% UB

1 -1.565833  2.371956 5.948790

2 2.414651  6.052777 9.696141

3 -7.784304 -2.816127 2.278009

Here, the matrix x provides the new values of adjusted temperature anomalies and SOI values in the forecast horizon of 3. When there are multiple independent series, x must be entered as a matrix, each row of which has h elements and corresponds to each independent series.

### Goodness-of-fit measures

There are three auxiliary functions included in dLagM: finiteDLMauto(), GoF(), and sortScore(). The GoF() function computes mean absolute error (MAE), mean squared error (MSE), mean percentage error (MPE), symmetric mean absolute percentage error (sMAPE), mean absolute percentage error (MAPE), mean absolute scaled error (MASE), mean relative absolute error (MRAE), geometric mean relative absolute error (GMRAE), mean bounded relative absolute error (MBRAE), unscaled MBRAE (UMBRAE) for distributed lag models using the formulations of Hyndman et al. [38, p. 26] and Chen et al. [29]. When there is only one model passed in the function, MBRAE and UMBRAE are not calculated. The function sortScore displays sorted AIC, BIC, MASE, MAPE, sMAPE, MRAE, GMRAE, or MBRAE scores in the ascending order to flag the best performing model easily.

The following code chunk computes the sorted MASE and other measures over the fitted DLMs in the previous sections.

> sortScore(x = MASE(dlmFit1, dlmFit2, KoyckFit, ARDLfit2), score = “mase”)

        n    MASE

ARDLfit2 1591 0.6561139

KoyckFit 1594 0.9399988

dlmFit2  1589 1.3694767

dlmFit1  1588 1.3702182

> GoF(dlmFit1, dlmFit2, KoyckFit, ARDLfit2)

       n    MAE    MPE   MAPE   sMAPE    MASE

dlmFit1 1588 3.131399 0.8207351 1.353352 1.6745267 1.3702182

dlmFit2 1589 3.133837 0.8035919 1.315302 1.6875275 1.3694767

KoyckFit 1594 2.161233 0.5037362 3.065148 1.0002000 0.9399988

ARDLfit2 1591 1.502217 0.3403850 2.367279 0.7693254 0.6561139

         MSE   MRAE   GMRAE   MBRAE   UMBRAE

dlmFit1 16.13457 7.890835 1.4464045 1.8943210 -2.1181668

dlmFit2 16.23229 8.740936 1.4243370 0.5903609 1.4411730

KoyckFit 7.85227 3.864511 0.9760243 0.4545904 0.8334846

ARDLfit2 3.80717 7.013070 0.6688625 0.1175530 0.1332126

### Specification of finite lag length

Specification of lag length for finite DLMs is an issue that needs to be elaborated. dLagM includes finiteDLMauto() function to help finding the best model according to goodness-of-fit (GOF) statistics. It automatically searches for the optimal model in terms of one of MASE, AIC, BIC, GMRAE, MBRAE, or adjusted R-square. It is capable of handling multiple independent series for searching over finite DLMs and it works with only one independent series for polynomial DLMs. The call to the function is as follows:

finiteDLMauto(formula, data, x, y, q.min = 1, q.max = 10,

        k.order = NULL, model.type = c(“dlm”,“poly”),

        error.type = c(“MASE”,“AIC”,“BIC”,“GMRAE”,

        “MBRAE”, “radj”), trace = FALSE)

The input structures for formula, data, x, and y arguments are the same as those for the dlm() function. The arguments q.min and q.max specify the bounds of lag order *q* for the search. When the model.type is set to poly, the arguments x and y must be used to send the independent and dependent series into the function. The function fits all finite linear or polynomial DLMs for all lag and/or polynomial orders and calculates or extract MASE, AIC, BIC, GMRAE, MBRAE, adjusted r-squared, and the p-value of Ljung-Box test from the model objects. Then, it sorts the models according to the desired measure. By this way, it displays the effect of lag and/or polynomial order on the error/GOF measures to help the user to determine the optimal value of orders of the model.

## ARDL bounds testing

ARDL bounds test developed by Pesaran et al. [12] is an efficient approach for the cointegration analysis. This test is frequently used by practitioners due to its advantages over the other cointegration analysis techniques in a vast variety of fields. Mainly, we do not need to have a stationary (*I*(0)) or difference stationary (*I*(1)) series to run the analysis with this approach. To observe the short and long-run dynamics of the series, we can straightforwardly derive unrestricted error correction model. Also, the ARDL bounds test can be used for relatively small samples [12, 39].

To formulate the ARDL bounds test, we rewrite Eq (1) in the following conditional error correction form:

$$\begin{array}{c} \begin{array}{cc} \Delta Y_{t}=\mu_{0} & +\alpha_{0}Y_{t-1}+\alpha_{1}X_{1,t-1}+\cdots +\alpha_{k}X_{k,t-1}+\sum_{i=1}^{q}\gamma_{i}\Delta Y_{t-i}\\ & +\sum_{j=0}^{p_{1}}\beta_{1,j}\Delta X_{1,t-j}+\cdots +\sum_{j=0}^{p_{k}}\beta_{k,j}\Delta X_{k,t-j}+e_{t}, \end{array} \end{array}$$ (2)

where *μ*_0_ is the intercept and Δ is the first difference of the series. The error correction part of the model, *EC*_*t*−1_ is

$$\begin{array}{c} EC_{t-1}=Y_{t-1}-\sum_{i=1}^{k}\frac{\alpha_{i}}{\alpha_{0}}X_{i,t-1}. \end{array}$$ (3)

The hypotheses of cointegration are written over the coefficients of the conditional error correction model given in Eq (2). Then, the test is applied with

$$\begin{array}{c} \begin{array}{c} H_{0}:\alpha_{0}=\alpha_{1}=\cdots =\alpha_{k}=0. \end{array} \end{array}$$ (4)

If *H*_0_ is rejected then we conclude the significance of cointegration between variables. A Wald test statistic is computed and compared to the asymptotic limits given by Pesaran et al. [12]. If the test statistic is lower than the given lower limit, *H*_0_ is not rejected and insignificance of cointegration between variables is concluded. If the test statistic is greater than the given upper limit, *H*_0_ is rejected and the significance of cointegration between variables is concluded. If the test statistic is in between the given lower and upper limits, no conclusion can be reached [12].

Pesaran et al. [12] define five different cases according to the incorporation of intercept (*μ*_0_) and trend (*μ*_1_) coefficients in the error correction term. Case 1: No intercept and no trend. Case 2: Restricted intercept and no trend. Case 3: Unrestricted intercept and no trend. Case 4: Unrestricted intercept and restricted trend. Case 5: Unrestricted intercept and unrestricted trend.

To implement the ARDL bounds test, the call to the ardlBound() function is

ardlBound(data = NULL, formula = NULL, case = 3, p = NULL,

      remove = NULL, autoOrder = FALSE, ic = c(“AIC”, “BIC”,

      “MASE”, “GMRAE”), max.p = 15, max.q = 15, ECM = TRUE,

      stability = TRUE)

The arguments data and formula are defined in the same way as the ardlDlm() function. The argument p shows the order of short-run response or it is specified as a data.frame to define a different order of short-run response for each variable. It is possible to drop different lags of dependent and independent series from the model by using remove argument which is a list of another list, p and vector, q. For example, to drop lags 1 and 2 of the first differences of the series X1 and X2 and drop the lags 2 and 5 of the dependent series, it is defined as remove = list(p = list( dX1 = c(2,4), dX2 = c(2,4)), q = c(2,5)). If ECM = TRUE, the error correction model corresponding to the case is also fitted and included in the output. If both ECM and stability is TRUE, the CUSUM and moving sum (MOSUM) of recursive residuals, and CUSUM of squared recursive residuals plots are generated using the package strucchange [40] to examine the stability of the residuals. If autoOrder is TRUE, the lag orders of the test are found by the function ardlBoundOrders(), which implements the proposed order specification algorithm explained in the next section. The ardlBound() function applies Breusch-Godfrey [41] and Ljung-Box [42] tests to detect autocorrelation in residuals, Breusch-Pagan [43] test to check against heteroskedasticity of residuals, Shapiro-Wilk’s [44] test for the normality of residuals, and Ramsey’s RESET [45] test to check the correctness of functional form of the model.

Asymptotic critical values (CVs) for the ARDL bounds test are given by Pesaran et al. [12]. Since these CVs are based on large samples, Narayan [46] provides small-sample CVs for Cases II—V. ardlBound() function calls pssbounds() function from nardl package [23] to get the CVs. This function is originally included in pss package and currently available in dynamac package [26]. For Cases II—V, pssbounds() function produces small-sample CVs in the brackets of 5 observations from 30 to 80 and produces asymptotic CVs for the sample sizes greater than 80. For Case I, asymptotic CVs are produced for all sample sizes.

### Specification of orders for ARDL bounds test

Selection of orders for ARDL bounds test has a direct impact on the resulting inferences. The number of possible orders quickly increases as the number of independent series (*k*), *p*_*i*_ and *q* increase. For instance, for *k* = 3 and *p*_1_ = *p*_2_ = *p*_3_ = *q* = 5, the total number of possible models is 1080. In some economic applications, the orders are determined by the theory. However, in other cases, the mainstream approach to find a suitable set of orders is based on searching over the full-set of possible models using AIC, BIC, HQIC (Hannan-Quinn IC), or adjusted r-square statistics. We call this approach “full-search” in the rest of this section. This approach is prone to the weaknesses of the used statistic, and its computer implementation is not quite time-efficient and troublesome for practitioners with limited programming skills. The mainstream econometric software EViews includes an add-in program to run the full-search approach [27, 47]. In dLagM package, in addition to the full-search, we implement a two-staged approach to the selection of the orders by the ardlBoundOrders() function. This function provides the users at all levels of software skills with a user-friendly tool for the specification of orders. The main advantages of the two-staged approach are that it utilizes MASE, and GMRAE in addition to AIC and BIC as the decision criterion for the search and it is more time-efficient when the values of *k*, *p*_*i*_, and *q* are large. This makes it possible to search with a larger maximum limit on the orders for long series. For advanced users, the proposed algorithm can be coded under any R package or software that fits ARDL models as an add-in program or package such as EViews.

The ardlBoundOrders() function computes the optimal lag structure for the short-run relationships and autoregressive part of the ARDL model for the ARDL bounds testing. The call to the function is as follows:

ardlBoundOrders(data = NULL, formula = NULL, ic = c(“AIC”, “BIC”,

         “MASE”, “GMRAE”), max.p = 15, max.q = 15,

         FullSearch = FALSE)

When FullSearch = TRUE, the full-search is implemented, and when it is set to FALSE, the two-staged search approach is implemented as in Algorithm 1.

**Algorithm 1**.

1. Assume all p-orders are equal (*p*_1_ = *p*_2_ = ⋯ = *p*_*k*_ = *p*), set *p* = 1 and *q* = 1.
2. Fit the conditional error correction model. Increase the value of *q* by one. Repeat this step until max.q is reached. Then, move on to the next step.
3. If *p* is less than or equal to max.p, increase the value of *p* by one and go to step 2. Otherwise, go to the next step.
4. Find the (p*, q*) pair that gives the optimal value to the stat.
5. Expand a grid with all combinations of *k* factors with p* levels.
6. Take each combination in the grid as the value of *p*_1_, *p*_2_, …, *p*_*k*_ and fit the conditional error correction model with orders (*p*_1_, *p*_2_, …, *p*_*k*_, q*).
7. Report the $\left(p_{1}^{*},p_{2}^{*},\ldots ,p_{k}^{*},q^{*}\right)$ combination that optimizes the stat as the optimal value or orders for the ARDL bounds test.

The first stage of Algorithm 1 is composed of the steps 1-3, where we find the number of autoregressive lags (*q*) that gives us a suitable value for the desired GOF statistic when all the orders of short-run relationships are assumed to be equal. After fixing the value of *q* at the first stage, we find the optimal combination of the p-orders at the second stage. To benchmark the computational efficiency of our approach with the full-search, suppose we have *k* = 3, max.p = 10 and max.q = 10 which define a moderate-sized search space. With the full-search approach, we need to fit 11^3^ ⋅ 10 = 13, 310 models. Whereas in our approach, we fit 11 ⋅ 10 = 110 models at the first stage. The number of models to be fitted at the second stage depends on the optimal value of *p*. In the worst-case scenario, we get *p* = max.p and fit 11^3^ = 1, 331 models at the second stage. In total, the maximum number of models to be fitted in our approach is 1,441 which is nearly 10% of the full-search approach. Therefore, in any platform it is implemented, our approach will run faster than the full-search.

The main drawback of our approach is the loss of information because of not searching the whole space of possible models. However, since we find an optimal value for *q* by assuming all *p*-orders are equal, this takes us to the proximity of the optimal set of lag orders in our search algorithm. Then, at the second stage, we fine-tune by allowing the *p*-orders to vary across the independent series in the short-run relationship part of the ARDL model. The full-search algorithm gives the optimal values of each GOF statistic as well as the corresponding lag orders to benchmark with the same values resulting from our algorithm to see the magnitude of information loss. To demonstrate this in practice, we run both full-search and proposed search approaches on 4 yearly, quarterly, monthly, and daily datasets and compare the values of GOF statistics and lag orders with *k* = 2, 3, and various settings of max.p and max.q. The following benchmarking code is implemented on a MacBook Pro computer with 2.9 GHz Intel Core i7 processor and 16 GB 2133 MHz LPDDR3 memory.

tic(“Standard”)

orders1<- ardlBoundOrders(data = seaLevelTempSOI, formula = formula1, ic = “AIC”, max.p = 10, max.q = 10, FullSearch = TRUE)

toc()

orders1$p; orders1$q; orders1$min.Stat

tic(“Proposed”)

orders2 <- ardlBoundOrders(data = seaLevelTempSOI, formula = formula1, ic = “AIC”, max.p = 10, max.q = 10)

toc()

orders2$p; orders2$q; orders2$min.Stat

We run this code chunk with AIC, BIC, MASE, and GMRAE statistics and record implementation times, optimal orders, and the value of the GOF statistics for the datasets and model formulas presented in Table 1. The results of trials with two independent series are reported in Table 2 and those with three independent series are presented in Table 3.

**Table 1:** Description of the test cases for the comparison of the full-search and the proposed algorithm for the specification of orders in the ARDL bounds testing.

Trial	Dataset	Package	Formula	n	Freq.	max.p	max.q
1	ineq	dynamac	concern ~ incshare10 + urate	49	1	5	5
2	M1Germany	dynlm	logm1 ~ loggnp + interest	147	4	7	7
3	M1Germany	dynlm	logm1 ~ loggnp + interest + logpricet	147	4	5	5
4	seaLevelTempSOI	dLagM	GMSL ~ LandOcean + SOI	1595	12	10	10
5	chicagoNMMAPS	dlnm	death ~ o3 + temp	5114	365	10	10

n: The number of observations in the dataset. Freq.: Frequency of the series.

**Table 2:** Benchmarking of p and q lag orders, implementation time, and the value of GOF statistic for the full-search and the proposed algorithm for Trials 1, 2, 4, and 5.

Trial	Full-Search algorithm						Proposed algorithm				
	Statistic	p		q	Time (sec)	Value	p		q	Time (sec)	Value
		*X*_1_	*X*_2_				*X*_1_	*X*_2_			
1	**AIC**	1	1	1	3.96	-247.3	1	1	1	1.64	-247.3
	**BIC**	1	1	1	3.67	-255.8	1	1	1	1.66	-255.8
	**MASE**	5	5	5	3.65	0.337	4	3	5	1.80	0.340
	**GMRAE**	4	3	5	3.59	0.239	4	3	5	1.79	0.239
2	**AIC**	4	4	4	13.01	-727.6	4	3	4	3.13	-725.6
	**BIC**	4	1	4	12.67	-677.1	4	1	4	4.71	-677.1
	**MASE**	5	7	7	13.07	0.124	5	7	7	4.66	0.124
	**GMRAE**	5	6	6	13.27	0.080	4	7	4	4.63	0.084
4	**AIC**	2	2	10	143.6	6413	1	1	10	15.69	6413
	**BIC**	1	1	10	140.2	6515	1	1	10	29.83	6515
	**MASE**	8	9	9	136.6	0.514	8	9	9	26.69	0.514
	**GMRAE**	4	8	8	137.9	0.509	5	3	8	17.01	0.512
5	**AIC**	5	4	5	35.81	40397	5	4	5	13.87	40397
	**BIC**	1	3	5	35.43	40509	1	3	5	13.62	40509
	**MASE**	5	4	5	34.27	0.435	5	4	5	13.15	0.435
	**GMRAE**	4	2	5	33.85	0.565	4	2	5	10.73	0.565

**Table 3:** Benchmarking of p and q lag orders, implementation time, and the value of GOF statistic for the full-search and the proposed algorithm for Trial 3.

Algorithm	Statistic	p			q	Time (sec)	Value
		*X*_1_	*X*_2_	*X*_3_			
Full Search	**AIC**	4	1	5	4	26.52	-729.78
	**BIC**	4	1	1	4	27.39	-671.25
	**MASE**	4	4	5	4	27.05	0.118
	**GMRAE**	2	1	5	4	26.64	0.077
Proposed	**AIC**	4	1	4	4	3.68	-729.26
	**BIC**	4	1	1	4	6.35	-671.25
	**MASE**	4	4	5	5	6.48	0.119
	**GMRAE**	3	3	1	4	2.39	0.079

For all GOF measures, as expected, our algorithm is 3 to 11 times faster than the full-search algorithm. When AIC is used as the target GOF measure, we get the same or more parsimonious orders with near-optimal AIC values via our search algorithm for all trials. For BIC, we find the same orders in all trials faster than the full-search algorithm. Targeting for MASE produces different lag orders in Trials 1 and 3. In Trial 1, the proposed algorithm recommends a similar but more parsimonious model with a very close MASE to its full-search counterpart. Similarly, the proposed algorithm identifies the same orders as the full-search approach quicker in Trials 1 and 5 with GMRAE. In Trials, 2, 4, and 5, minimizing GMRAE returns more parsimonious models with the proposed algorithm. Overall, this benchmarking shows us that the information loss with the two-stage algorithm is negligible while the gain in computational time is highly significant.

The full-search approach gives optimal orders in terms of the GOF measures. However, optimising the GOF measures and simultaneously keeping the model as parsimonious as possible is challenging and can be done by a trial-and-error method if the user is experienced. The values of GOF measures obtained by our search algorithm is very close to the optimal values by the full-search and the proposed orders constitute more parsimonious models in a computationally efficient way. Another consideration is that the trial-and-error method cannot readily be embedded in artificial intelligence algorithms such as recommender systems. In this sense, our algorithm is useful and efficient when it is embedded in self-learning systems to specify the orders automatically. Also, it provides less experienced users with an efficient tool for order specification.

The dLagM package can be compared to EViews and dynamac packages in terms of its functionality for ARDL bounds testing. We summarize this comparison as follows:

- The main distinction of dLagM is that it implements both the full-search and the proposed algorithms to optimize AIC, BIC, MASE, or GMRAE, and reports the optimized orders for ARDL bounds testing. Both EViews and dynamac do not offer any built-in functions for this aim by default. There is an add-in program available for Eviews to implement the full-search which is limited to a maximum of 10 lags [47].
- dLagM readily displays Breusch-Godfrey, Ljung-Box, Breusch-Pagan, Shapiro-Wilk’s, and Ramsey’s RESET tests as residual diagnostics, and recursive CUSUM, recursive CUSUM of Squares, and recursive MOSUM plots as stability diagnostics. EViews has Jarque-Bera, Breusch-Pagan-Godfrey Serial Correlation LM, Harvey, Glejser, ARCH, and White heteroskedasticity tests as residual diagnostics, and recursive CUSUM, recursive CUSUM of squares, recursive coefficients, N-step forecast tests, and Ramsey’s RESET tests as stability diagnostics. dynamac implements Breusch-Godfrey, Chi-square, and F tests can be implemented by a separate function call. The dynamac::pssbounds() function only displays the F-statistic and critical values for the test.
- As input dLagM needs the lag orders and dataset to run the ARDL bounds test. This is similar in EViews. Whereas dynamac requires fitting the right ARDL model outside the dynamac::pssbounds() function.

Overall, as open-source software, dLagM has almost a similar functionality for ARDL bounds testing as EViews plus some different diagnostic tests and an alternative approach to the specification of orders. It is very easy to hook up the dLagM to other research software in R or other software such as Matlab and Python that can communicate with R due to dLagM’s input/output interface and R’s functionality.

### Illustration of ARDL bounds testing with dLagM package

To illustrate the use of the package for ARDL bounds testing, we will investigate short and long-term relationships between change in the sea level (relative to 1993-2008 average), land and ocean temperature anomalies and SOI provided by the seaLevelTempSOI dataset.

In practice, we have to check if the signals in both series are significant or white noise by using the rolCorPlot() function and we need to check the existence of a spurious correlation between the dependent and independent series. Since our aim here is to illustrate the use of the ardlBound() function, we do not present these preliminary analyses here. They are included in the R scripts given in the “Supporting information” section. We specify lag orders using ardlBoundOrders() function as follows:

> orders2 <- ardlBoundOrders(data = seaLevelTempSOI, formula = formula1, ic = “BIC”, max.p = 10, max.q = 10)

> orders2$p

 LandOcean SOI

1      1 1

> orders2$q

[1] 10

From this analysis, we get *p*_1_ = *p*_2_ = 1, *q* = 10. We put these values into a data.frame object to pass on the main function. Some software starts the summations in Eq (2) from 0. However, since we start them from 1 following Pesaran et al. [12], we add 1 to the elements of the data.frame object:

> p <- data.frame(orders2$q, orders2$p) + 1

Then, we run the ARDL bounds test by the following call:

> test1 <- ardlBound(data = seaLevelTempSOI, formula = formula1, case = 1, p = p, ECM = TRUE)

Here, we fit the model with no intercept and no trend represented by case = 1. Since the full output of this call is too long, we leave running the codes given in “Supporting information” section and getting the full output to readers. The output shows us the Breusch-Godfrey, Ljung-Box, Breusch-Pagan, Shapiro-Wilk’s, and Ramsey’s RESET tests as residual diagnostics along with the results of the ARDL bound test. We get non-normal residuals and significant autocorrelations in the residuals by the Shapiro-Wilk and Breusch-Godfrey tests, respectively. RESET test indicates that there is no model specification issue. Since we find an F-statistic of 118.67, which is greater than all the critical values for all *α*-levels, we conclude the significance of cointegration between the change sea level, land and ocean temperature anomalies, and SOI. Then, the output shows the coefficient estimates and significance tests for the error correction model. The error correction coefficient is negative (-0.9412) as expected and highly significant (p-value < 2e-16), which is consistent with the ARDL bounds test. This means that there is a significant long-run relationship or cointegration between the change in sea levels and the exploratory series. The last bit of the output shows the values of the long-run coefficients for the land-ocean temperature anomalies and SOI. To get the significance tests for the long-run coefficients, we use summary(test1$model$modelFull).

**Fig 2:**
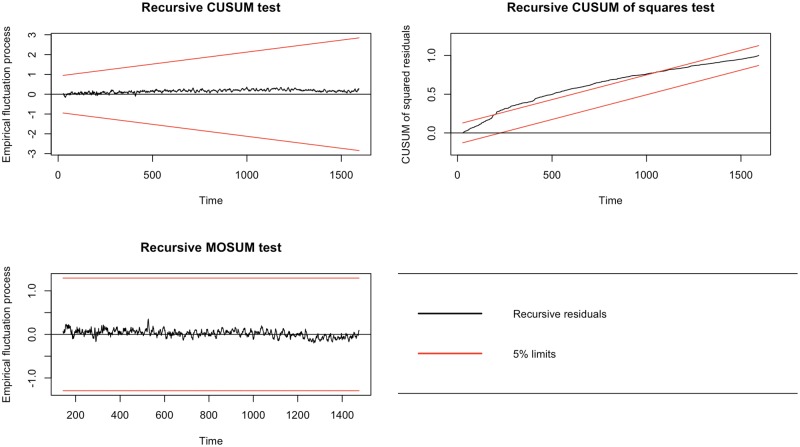
Recursive CUSUM, CUSUM of squares and MOSUM plots for the model stability.

The stability of the model is another important issue to check in ARDL bounds testing. If both ECM = TRUE and stability = TRUE, the cumulative sum (CUSUM), CUSUM of squares, and MOSUM charts [48, p. 213] [40] are generated using recursive residuals. While the recursive CUSUM, recursive CUSUM of squares plots are also generated by EViews, dLagM package provides recursive MOSUM chart to give more insight into the investigation of model stability issue in ARDL bounds testing. Recursive CUSUM is produced over all previous observations, whereas recursive MOSUM uses a rolling window of previous observations [48]. So, MOSUM is more sensitive to local departures from stability. Recursive MOSUM chart requires a relatively long series to be displayed. Recursive CUSUM of squares can be used to identify instability in the variances of coefficients over time. If there is a structural change in the volatility of the model coefficients, we expect the recursive CUSUM of squares cross the limits. Fig 2 displays the recursive CUSUM, recursive CUSUM of squares, and recursive MOSUM plots for the example.

Since the recursive CUSUM and MOSUM residuals remain close to 0 without fluctuating outside the 5% limits, there is no sign of change in the coefficients over time. However, recursive CUSUM of squares test indicates that there is an instability in the volatility of coefficients as recursive CUSUM of squares goes outside the 5% limits. This is a good example of instability in the volatility of coefficients while they are stable over time.

Overall, for this example, the model specification should be changed to get acceptable diagnostic results before proceeding with the decision provided by the ARDL bounds test. Considering that the orders used for the test are minimizing BIC among all possible models with max.p = 10 and max.q = 10, minimizing the GOF measures to specify the orders does not necessarily ensure getting stable results. Therefore, it is crucial to check residual and stability diagnostics.

## Discussion

Distributed lag models (DLMs) constitute a widely used class of regression models for modelling time series data by including independent time series into the model. Although the primary field of application for DLMs is econometrics, it is also frequently used in other areas like agriculture, environmental analyses, health, and population studies. In this article, we propose a fast algorithm for the selection of lag orders for ARDL bounds testing and describe the functionality of the package dLagM developed for implementation of finite linear, polynomial, Koyck, autoregressive DLMs, and the ARDL bounds testing for cointegration. The package also includes useful functions to find the orders of finite and polynomial DLMs, test the significance of signals using rolling correlations, compute and sort goodness-of-fit measures (GOF) AIC, BIC, MAE, MSE, MPE, sMAPE, MAPE, MASE, MRAE, GMRAE, MBRAE, and recently proposed UMBRAE for distributed lag models. In this sense, the package is capable of computing a wide-range of GOF measures. One of the main contributions of the package is that it implements a new search algorithm to specify the lag orders for ARDL bounds testing. As an advantage over the existing software for ARDL bounds testing, the new algorithm provides a significant improvement in computation time to specify lag orders while approximating to the optimal value of AIC, BIC, MASE, or GMRAE.

Both input and output structures of the functions in the dLagM package are very similar to those of the mainstream functions of R and it does not require any preprocessing of data. For polynomial and Koyck transformations, estimates of original parameters before the transformation are also computed and displayed to enrich the inferences. Another beneficial functionality of the package is that it allows model fitting with specific lags of independent series for finite DLMs and those of both dependent and independent series in autoregressive DLMs rather than fitting only full models. Beyond its use in the data analysis, since the dLagM package is a part of R, it is very easy to inject the functionality of package into other open-source research software. As a readily available, open-source software, these features make the dLagM package flexible and user-friendly; hence, the package is useful for a broad audience.

One of the limitations of the package is that it handles only one independent series for polynomial DLM and Koyck models. One of the near-future improvements of the package is to include multiple independent series for these models.

## Supporting information

S1 Code R scripts of analysis. All the scripts used to implement analyses are given in the supporting electronic document available at https://doi.org/10.1371/journal.pone.0228812.
(R) Click here for additional data file.
